# A cyclic peptide retards the proliferation of DU145 prostate cancer cells in vitro and in vivo through inhibition of FGFR2

**DOI:** 10.1002/mco2.48

**Published:** 2020-12-14

**Authors:** Yibo Zhang, Man Ouyang, Hailong Wang, Bihui Zhang, Wenhua Guang, Ruiwu Liu, Xiaocen Li, Tsung‐Chieh Shih, Zhixin Li, Jieqiong Cao, Qiling Meng, Zijian Su, Jinshao Ye, Feng Liu, An Hong, Xiaojia Chen

**Affiliations:** ^1^ Department of Cell Biology, College of Life Science and Technology, Jinan University National Engineering Research Center of Genetic Medicine Guangdong Provincial Key Laboratory of Bioengineering Medicine Guangdong Provincial Biotechnology Drug & Engineering Technology Research Center Jinan University Guangzhou China; ^2^ Department of Biochemistry and Molecular Medicine University of California Davis Sacramento California; ^3^ Guangdong Key Laboratory of Environmental Pollution and Health School of Environment Jinan University Guangzhou China; ^4^ China Nuclear Power Technology Research Institute Co Ltd Shenzhen China

**Keywords:** cancer therapy, cyclic peptide, fibroblast growth factor receptor 2 (FGFR2), peptide drug

## Abstract

In malignancies, fibroblast growth factor receptors (FGFRs) signaling is reinforced through overexpression of fibroblast growth factors (FGFs) or their receptors. FGFR2 has been proposed as a target for cancer therapy, because both the expression and activation of FGFR2 are boosted in various malignant carcinomas. Although several chemicals have been designed against FGFR2, they did not exhibit enough specificity and might bring potential accumulated toxicity. In this study, we developed an epitope peptide (P5) and its cyclic derivative (DcP5) based on the structure of FGF2 to limit the activation of FGFR2. The anticancer activities of P5 and DcP5 were examined in vitro and in vivo. Our results demonstrated that P5 significantly inhibited the cell proliferation in FGFR2‐dependent manner in DU145 cells and retarded tumor growth in DU145 xenograft model with negligible toxicity toward normal organs. Further investigations found that the Gln4 and Glu6 residues of P5 bind to FGFR2 to abolish its activation. Moreover, we developed the P5 cyclic derivative, DcP5, which achieved reinforced stability and anticancer activity in vivo. Our findings suggest P5 and its cyclic derivative DcP5 as potential candidates for anticancer therapy.

## Graphical abstract

In various malignancies, fibroblast growth factor receptors (FGFRs) signaling is reinforced, resulting in tumor progression and poor prognosis. Herein, we developed an FGFR2 competitive inhibitory peptide (P5) to limit the overactivation of FGFR2 signaling. Moreover, this P5 peptide was upgraded to DcP5 through cyclization to achieve improved in vivo stability and FGFR2‐binding affinity.

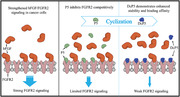

## 1 INTRODUCTION

Malignancies cause millions of global deaths every year. Many strategies, including surgery, radiotherapy, and chemotherapy, have been employed to improve the overall survival of these patients. However, there are still many patients suffering from side effects of these therapeutic treatments. Recently, targeted therapy was highlighted for its excellent selectivity. These drugs demonstrated precise inhibitory effect on the signaling molecules that are specifically amplified and essential in cancer cells; for instance, receptor tyrosine kinases (RTKs), a family of receptors overexpressed in various cancer cells and crucial for cell proliferation, apoptosis, drug resistance, metastasis, and angiogenesis.^1^, ^2^

As a group of RTKs family, fibroblast growth factor receptors (FGFRs) play critical roles in many key behaviors, including cell proliferation, migration, differentiation, and survival. As their ligands, FGFs including FGF1 (acidic FGF), FGF2 (basic FGF), FGF3‐6, FGF7, FGF8‐10, and FGF16‐23 bind to different FGFRs and activate their corresponding receptors.^3^ However, altered FGFs/FGFRs signaling could result in pathogenesis. Usually, the FGF/FGFR signaling was amplified in various malignancies. For instance, Deng et al demonstrated that nearly half of gastric cancers demonstrated amplified FGFR2 signaling.^4^ Chen et al found that FGFR2 expression level and ligand‐induced phosphorylation are responsible for the progression and poor prognosis in various cancers.^5^ These highlighted FGFR2 signaling as a potential cancer therapeutic target.

Great efforts have been attempted to limit FGFR signaling for cancer therapy. Recently, several small chemical molecules‐based FGFR inhibitors have been designed with remarkable anticancer activities in preclinical investigations.^6^ For instance, FGFR1/2/3 inhibitor AZD4547,^7^ FGFR4 inhibitor BLU9931,^8^ and FGFR1 inhibitor UPR1376^9^ have been demonstrated to be potential anticancer reagents. However, there are still no FGFR inhibitors approved for clinical cancer therapy. One of the major reasons is that although these small chemical molecules‐based FGFR inhibitors exhibited considerable activities, they showed cross‐binding activities among FGFR family members, and even other RTKs, which therefore brought terrible side effects. Moreover, the metabolism and clearance of such molecules remains unclear, and shows interpersonal differences.^10^, ^11^, ^12^ Therefore, it is of significant importance to develop FGFR inhibitors with greater selectivity and lower toxicity.

Peptide‐based drugs are catching more and more attention for their unique molecular properties.^13^ They showed lower immunogenicity but similar specificity when compared with the full‐length protein. However, peptides demonstrated greater selectivity than small chemical molecules. Moreover, peptides could be degraded into amino acids in vivo, which are totally nontoxic.^14^, ^15^ These properties allow peptides to be excellent candidates for cancer‐targeted therapy, such as FGFR2 inhibitors. As a drawback, the stability of peptides is still needs improvement. Cyclization is one of the major strategies to promote the stability of peptides. It has been employed to upgrade many instable peptides, and thus, cyclic peptides are attracting considerable interest owing to their efficiency in maintaining biostability within the proper range.^16^ According to the statistics, over 40 cyclic peptide drugs have been approved for clinical application, and 20 more are under clinical development.^17^ Herein, we developed an epitope peptide (P5) with potential affinity for the FGF2 binding site on FGFR2. Cyclization was employed to improve the stability of P5. Both P5 and its cyclic derivative, DcP5 demonstrated inhibitory effect on FGFR2. The anticancer activities of P5 and DcP5 were examined in vitro and in vivo.

## 2 RESULTS

### 2.1 Structure‐based peptide design

As the first step toward this study, we confirmed FGFR2 expression in different cancer cell lines by using both QPCR and Western blotting. The results indicated that FGFR2 is widely expressed in various cancer cells (Figure S1), suggesting FGFR2 as a target for drug design. Using the protein‐ligand docking simulation procedure, six peptide candidates were developed from the original sequence of FGF2 and FGFR2. The docking models of FGF2 and FGFR2 are shown in Figure S2A. The binding sites and regions of the six peptide candidates are shown in Figure S2B‐F.

**Figure 1:**
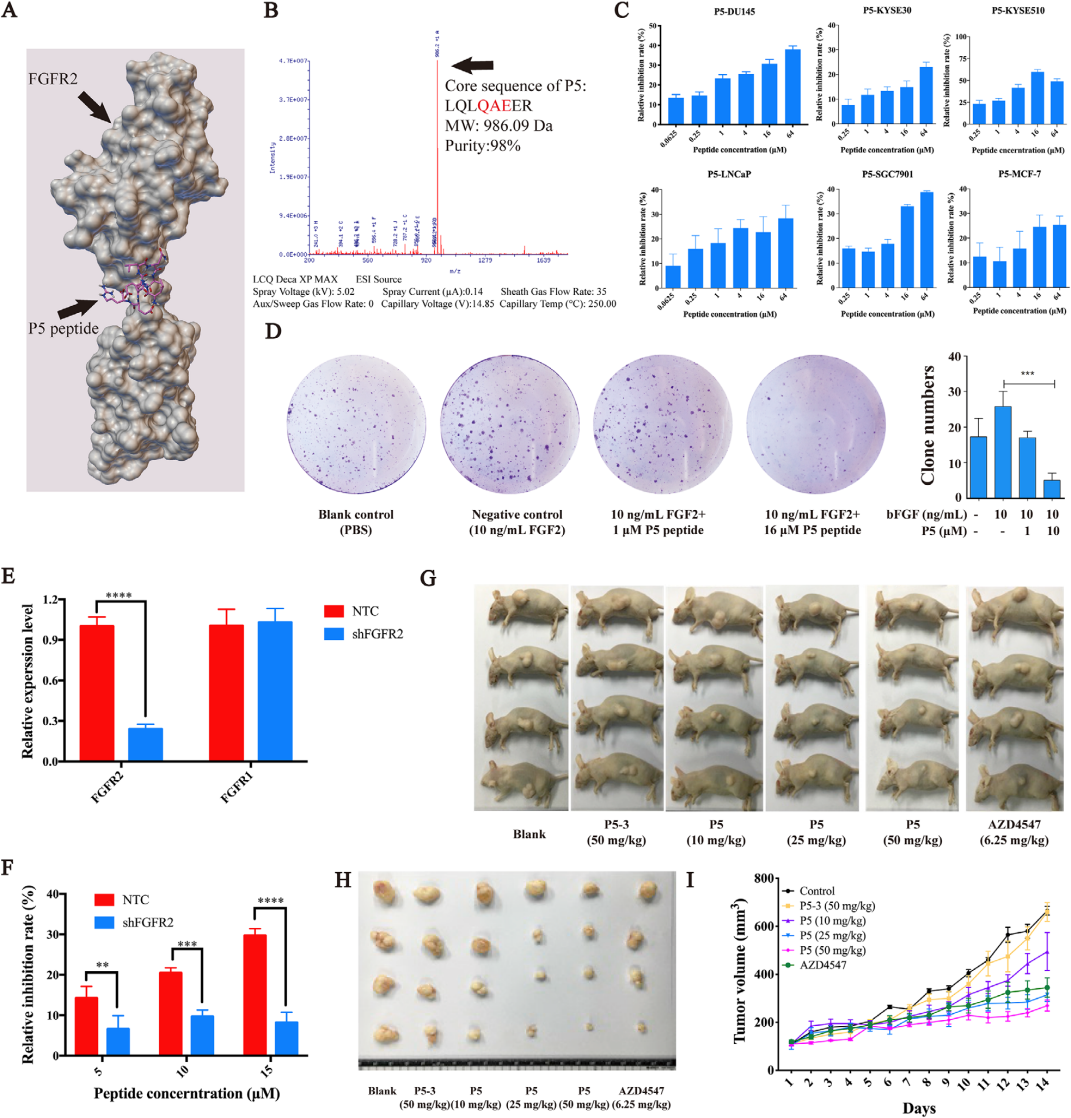
Peptide design and the anticancer activities of P5 in vitro and in vivo. A, Binding position of FGF2 and FGFR2, and the location of peptide P5 is tinted with red. B, MS spectrum of P5 peptide. C, Proliferation of DU145, KYSE30, KYSE510, MCF‐7, and SGC7901 cells exposed to different doses of P5 peptide. D, Colony formation of DU145 cells exposed to P5 with or without the presence of FGF2. E, The mRNA level of FGFR2 and FGFR1 in DU145 cells treated with NTC and shFGFR2 plasmid by qPCR; the housekeeping gene GADPH was employed as loading control. F, Growth‐inhibition activities of P5 in wild type and FGFR2‐knockdown DU145 cells. G, Typical images of DU145‐bearing null mice with different doses of P5 treatment. H, Typical images of DU145‐based tumors with different doses of P5 treatment. I, Quantification of the tumor volume in DU145‐based xenograft exposed to different doses of P5. *P < .05, **P < .01, ***P < .001, ****P < .0001

The physical interaction capability to FGFR2 of these docking candidates was subsequently evaluated by employing ITC assay. According to the ITC results, candidate P5 (LQLQAEER, amino acids 53‐60 of FGF2) demonstrated strong binding activity (KD = 4.90 × 10^‐7^ M) to FGFR2, while other candidates failed to bind to FGFR2 (data not shown). The binding model of the P5 peptide is shown in Figure 1A; and the P5 peptide was synthesized and confirmed by mass spectrometry (MS) (Figure 1B).

### 2.2 Dose‐dependent anticancer activities of P5 via competitive inhibition of FGFR2

To evaluate anticancer activity of P5, we measured the cell proliferation of DU145, KYSE30, KYSE510, MCF‐7, and SGC7901 cancer cell lines exposed to different doses of P5. The results demonstrated that the P5 peptide inhibits the proliferation of all these five cancer cell lines, especially DU145 cells (Figure 1C). Considering that P5 showed best growth‐inhibition activity in DU145 cells, further studies were performed in DU145 cells. To investigate proliferation inhibitory effect of P5, colony formation was evaluated for P5 with the presence of FGF2. As shown in Figure 1D, the P5 peptide abolished FGF2‐induced cell proliferation in a dose‐dependent manner. FGF2‐induced cell proliferation slightly reduced with the presence of 1 μM P5 peptide, while the cell proliferation was sharply blocked when the concentration of P5 increased to 16 μM. Our results suggested P5 as an inhibitor to antagonize FGFR2 signaling. We also measured the toxicity of P5 toward normal cells and the results demonstrated that P5 exhibited negligible toxicity in L02 normal liver cells, suggesting the safety of P5 in vitro (Figure S2C).

To study the role of FGFR2 in growth inhibition by P5, we attempted to knockdown FGFR2 by employing shRNA. As shown in Figure 1E, the relative expression level of FGFR2 reduced to about 30% of the negative control (NTC) group, while the mRNA level of FGFR1 remained comparable with the NTC group. Using this shRNA, we evaluated the growth‐inhibition activity of P5 in FGFR2 knockdown DU145 cells. The results demonstrated that the growth inhibitory effect of P5 significantly reduced in FGFR2 knockdown DU145 cells compared to that in wild type cells (Figure 1F). This indicates that P5 inhibits the growth of DU145 cells in an FGFR2‐dependent manner.

To further investigate the in vivo anticancer activity of P5 peptide, xenograft (DU145 cells) was employed, and results showed that P5 peptide significantly inhibits the tumor growth in dose‐dependent manner (Figure 1G‐I). In this experiment, one of the Pan‐FGFR inhibitors, AZD4547, was introduced as a positive control. Interestingly, although 6.25 mg/kg AZD4547 showed comparable anticancer activity with 10 mg/kg P5 peptide, P5 peptide has twice the molecular weight than AZD4547, indicating that P5 exhibits more excellent anticancer activity in vivo than AZD4547 at the same molarity. Moreover, H&E staining was performed for paraffin sections of major organs in the tumor‐bearing mice treated with P5 and AZD4547. Figure S3A shows the typical morphologies of organs in the mice exposed to P5 and AZD4546. We can observe that both P5 and AZD4547 demonstrated negligible toxicity toward heart, liver, spleen, lung, and kidney at their functional doses. Our results indicated that P5 is an excellent candidate for cancer therapy with low toxicity in vivo.

### 2.3 Gln4 and Glu6 are crucial for binding of P5 to FGFR2

**Figure 2:**
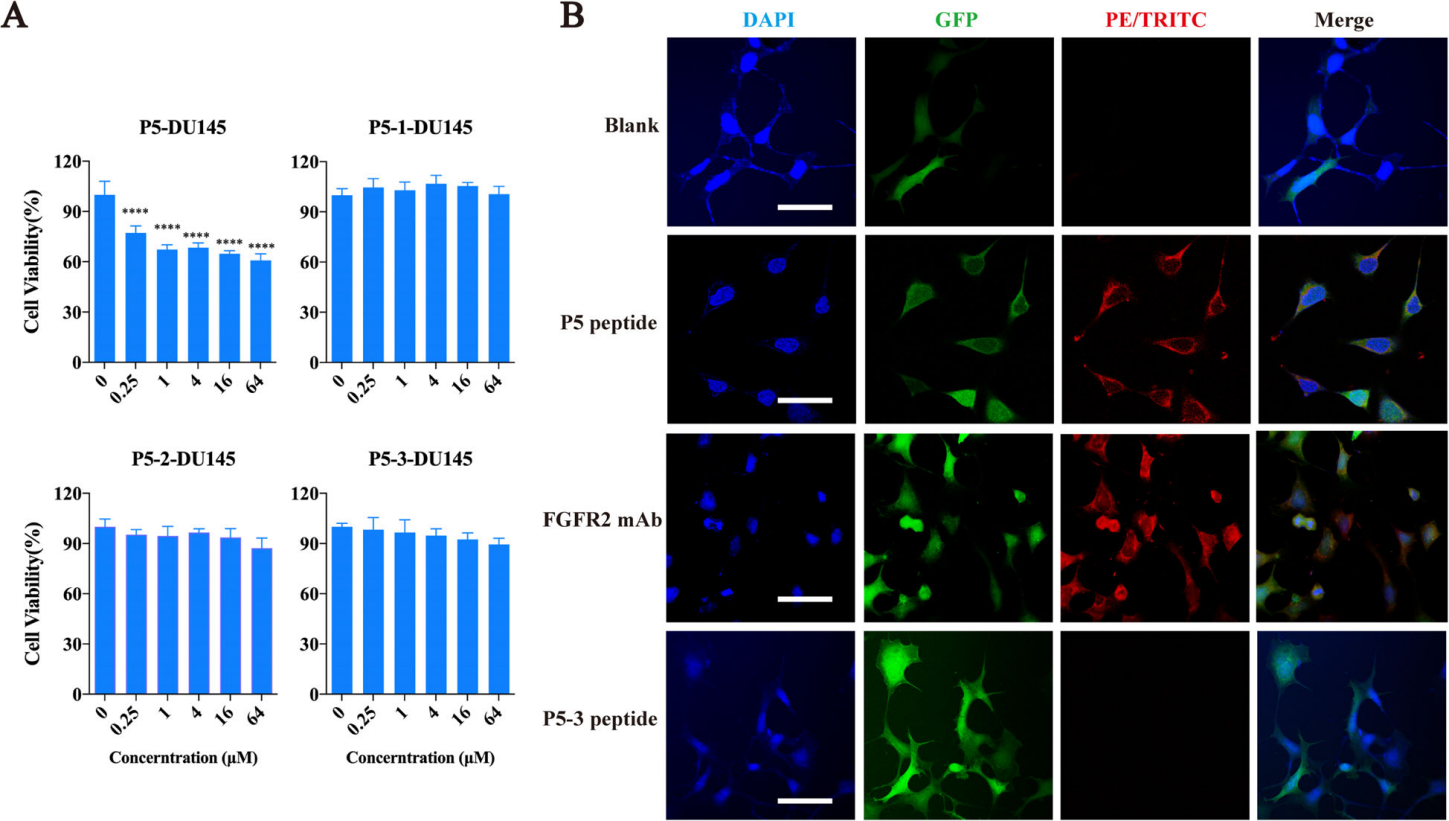
Growth inhibition activities of P5 is dependent on the binding affinity of P5 to DU145 cells. (A) Lost of growth inhibition activities of the P5 analogs, including P5‐1, P5‐2, and P5‐3. * refers to p<0.05, ** refers to p<0.01, *** refers to p<0.001 and **** refers to p<0.0001; (B) Indirect immunofluorescence asay (IFA) for P5 peptide and its epitope mutated analog (P5‐3) on the surface of DU145 cells (Bar=50 μm). Cells was treated with biotin‐labeled P5 and P5‐3 peptides, followed by the PE‐TRITC fluorescein (Red) ‐labeled streptavidin, which recognizes biotin specifically, while PE‐TRITC‐labeled secondary antibody was employed to recognize FGFR2 mAb as positive control group. GFP and DAPI were employed to indicate the location of DU145 cells

Considering inhibitory effect of P5 for FGFR2, we attempted to clarify how P5 inhibits FGFR2. Using docking simulation between P5 and FGFR2, we found that hydrophobic interactions of Gln4 and Glu6 are crucial in the ligand‐binding regions. To verify the binding site between P5 peptide and FGFR2, we designed three epitope analogs. Among these analogs, Gln4 was replaced by Ala in P5‐1 (LQLAAEER), Gln6 was replaced by Ala in P5‐2 (LQLQAAER), and both Gln4 and Glu6 were replaced by Ala in P5‐3 (LQLAAAER). They were chemically synthesized and subjected to a proliferation‐inhibition experiment in the DU145 cell line. As expected, the growth‐inhibition effect disappeared when either Gln4 or Glu6 was replaced by Ala (Figure 2A). Immunofluorescence assay (IFA) was employed to verify the difference in binding affinities between P5 and its analogs. The results of IFA indicated that the P5 peptide could remain on the cell, which was similar to the FGFR2 antibody (positive control), whereas the mutant P5‐3 peptide did not. We deduced that this is due to the binding failure of the epitope in P5‐3, which changed the affinity of P5‐3 peptide to FGFR2 (Figure 2B). Moreover, we measured the epitope‐mutated P5‐3 peptide in the xenograft model, and results demonstrated that there was no significant difference between control group and P5‐3 treatment group (*P *> .05), while P5 peptide significantly inhibited the tumor growth (Figure 1G‐I), indicating that replacement of the active amino acids affected the activity of the P5 peptide in vivo. These results, collectively, demonstrated that the binding of Gln4 and Glu6 in P5 peptide to FGFR2 abolished the activation of this receptor both in vitro and in vivo.

### 2.4 Cyclization enhances the FGFR2‐binding affinity and in vivo stability of P5 peptide

**Figure 3:**
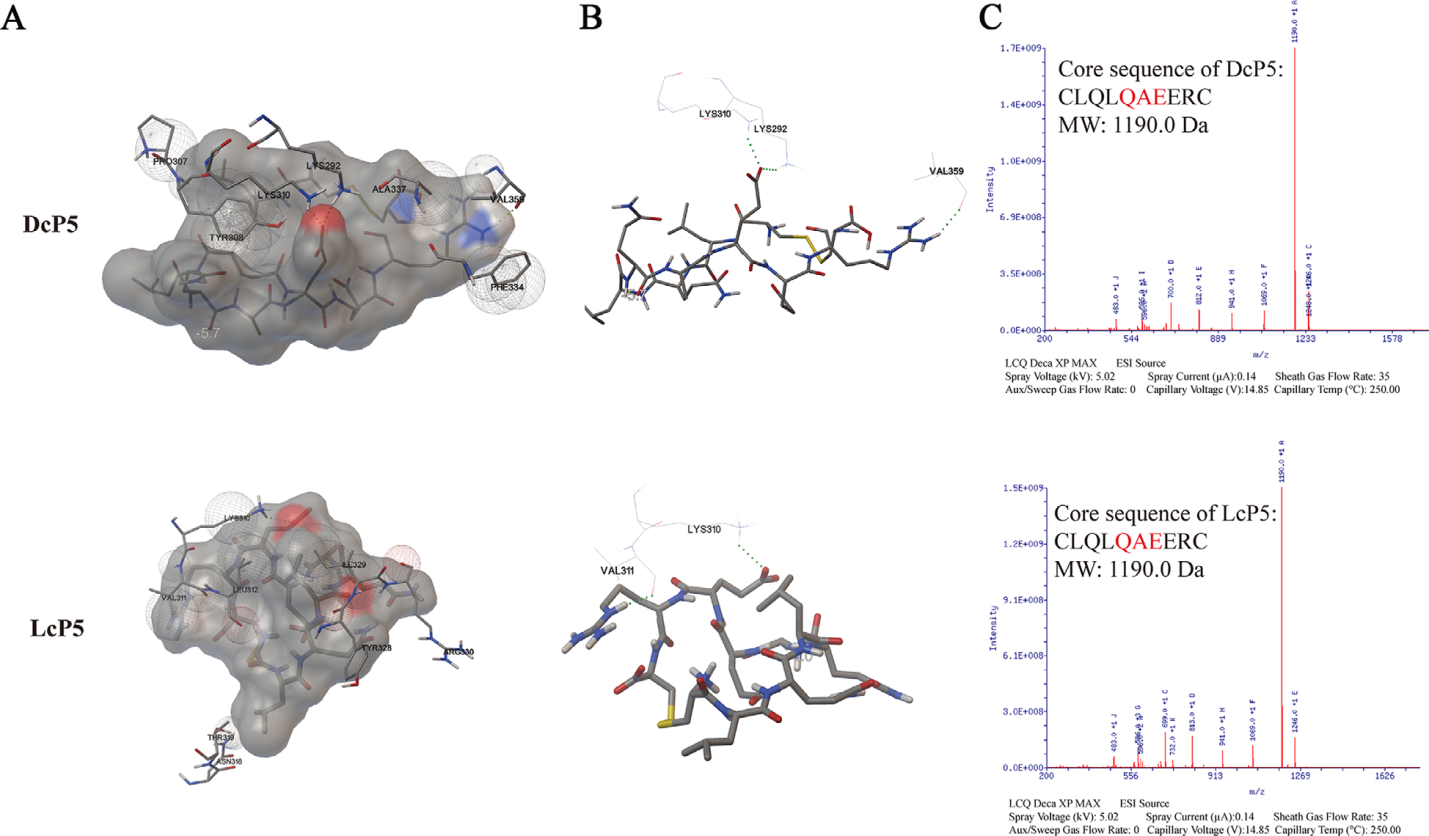
Cyclic peptide models and their MS spectra. A, Structures of DcP5 and LcP5 in surface view. B, Structures of DcP5 and LcP5 in stick model view. C, MS spectra of DcP5 and LcP5 cyclic peptides

**Figure 4:**
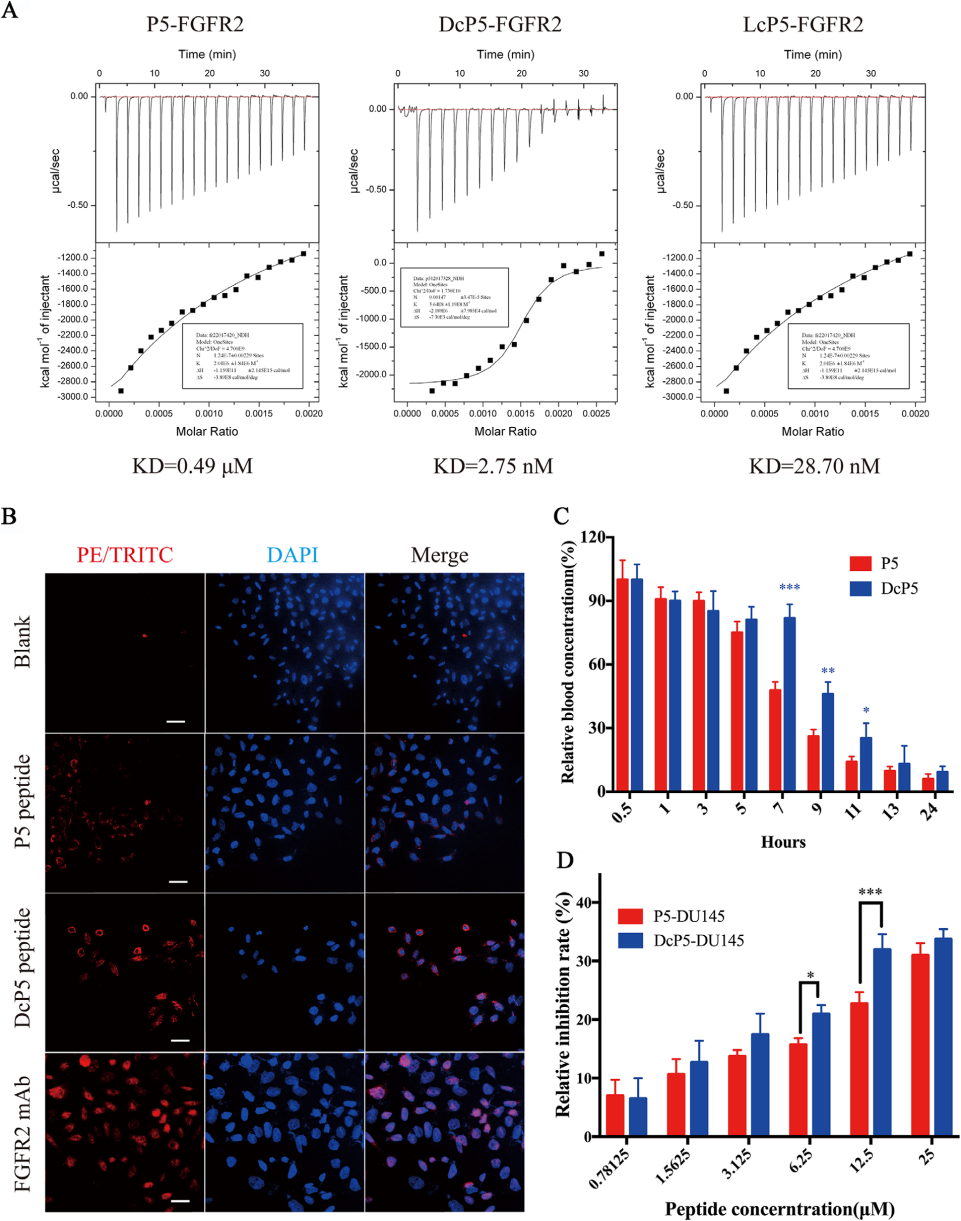
Cyclization improves the binding affinity and in vivo stability and in vitro growth inhibition activities of P5. (A) Binding affinity analysis of P5, LcP5 and DcP5 to FGFR2 by using ITC; (B) Binding affinities of P5 and DcP5 to DU145 by using IFA. Bar=50 μm. Cells was treated with biotin‐labeled P5 and P5‐3 peptides, followed by the PE‐TRITC fluorescein (Red) ‐labeled streptavidin, which recognizes biotin specifically, while PE‐TRITC‐labeled secondary antibody was employed to recognize FGFR2 mAb as positive control group. DAPI was employed to indicate the location of DU145 cells; (C) Quantification of in vivo P5and DcP5 peptide concentrations from 0.5 h to 24 h post‐administration; (D) Growth inhibition activities of P5 and DcP5 in Du145 cells. * refers to p<0.05, ** refers to p<0.01 and *** refers to p<0.001

To enhance the stability in vivo and anticancer activity of P5, we attempted to cyclize P5 peptide. The cyclization was achieved by adding a cysteine residue to each end of the P5 peptide and then forming a disulfide bond. Since cysteine is a chiral molecule, we used l‐cysteine and d‐cysteine to cyclize the P5 peptide and named them LcP5 and DcP5, respectively. The structures of DcP5 and LcP5 are shown in Figure 3A,B. The cyclic peptides were chemically synthesized and then verified by employing MS. As shown in Figure 3C, molecular weights of both DcP5 and LcP5 are 1190.38 Da, with purity of 98% (Figure S4). ITC analysis was performed to determine the changes in affinity due to the structural changes. According to the ITC results, DcP5 and LcP5 demonstrated even stronger affinities to the FGFR2 than the linear peptide P5 (Figure 4A and Figure S5). The affinity constant (KD) of DcP5 was 2.75 × 10^‐9^ M, while for LcP5 it was 2.87 × 10^‐8^ M. Therefore, our results reflected that DcP5 demonstrated the strongest affinity to FGFR2 and was selected for further investigation. Based on these findings, we hypothesized that DcP5 would exhibit better binding affinity to FGFR2 expressed on the cells, as well as anticancer activity than P5.

To test the above hypothesis, we verified binding affinity of the cyclic peptides to FGFR2 and the inhibitory effect on cell proliferation. Our results showed that both linear peptide P5 and cyclic peptide DcP5 bind to FGFR2 (Figure 4B). However, the fluorescent intensity in the cells treated by DcP5 was significantly stronger than that treated by P5. This difference is in accordance with the ITC result, indicating that the binding affinity at the cellular level is consistent with that at the molecular level.

To verify whether peptide cyclization affected the in vivo stability, we examined the peptide concentrations from 0.5 to 24 hours postadministration. As shown in Figure 4C, the concentrations of DcP5 and P5 in the peripheral blood decreased continuously during the period of 0.5‐24 hours after administration of the same dose (25 mg/kg). However, the concentration of DcP5 was twice than P5 0.5 hour after administration. Moreover, DcP5 was maintained for 7 hours before the blood concentration dropped below half *C*_max_, while P5 maintained the concentration of half *C*_max_ for 5 hours. Collectively, these results indicated that cyclization significantly enhanced the FGFR2‐binding affinity and in vivo stability of P5.

### 2.5 Cyclization of P5 peptide improves its anticancer activity

**Figure 5:**
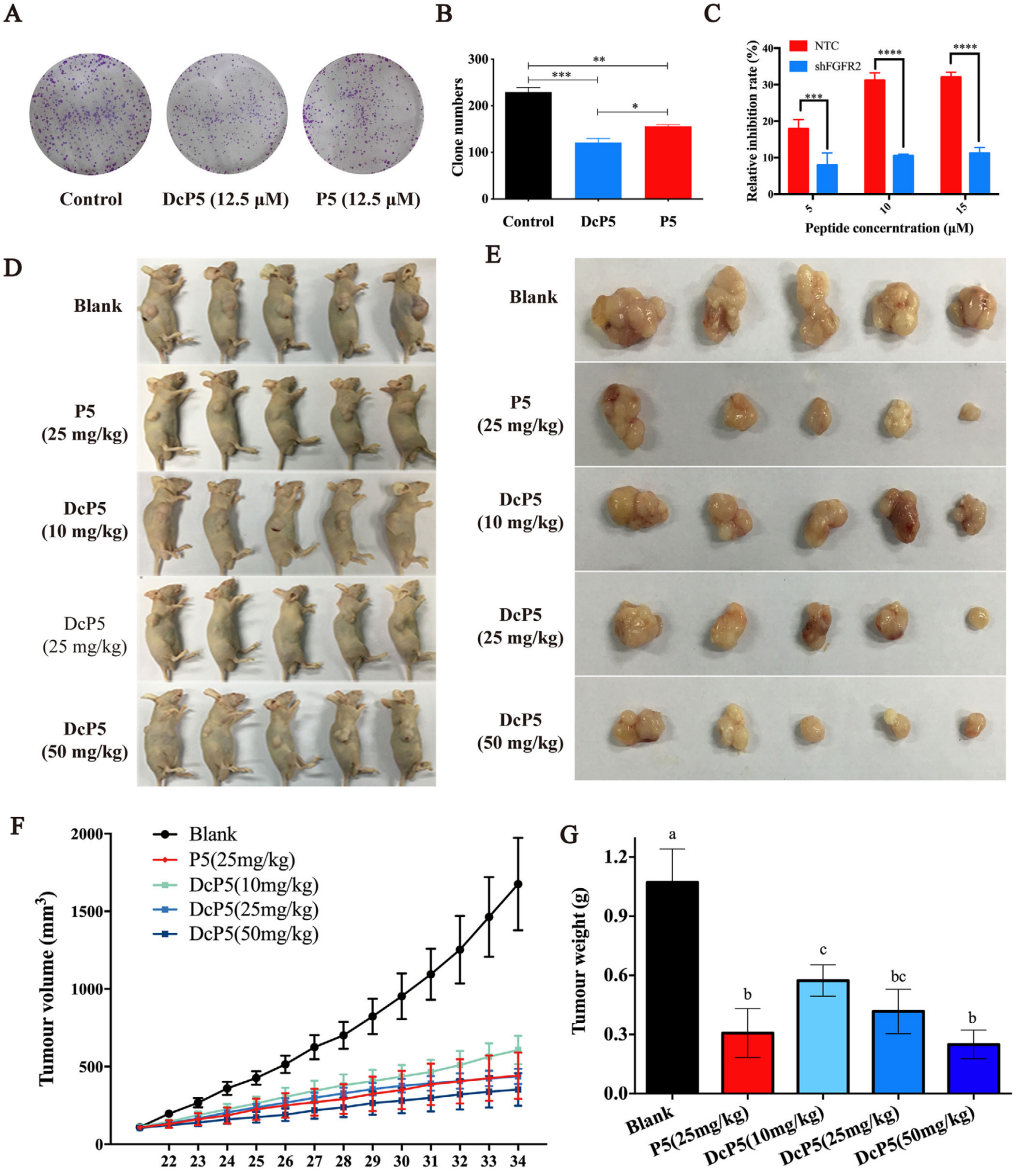
Cyclization enhances the anticancer activity of P5 in vitro and in vivo. A, Typical images demonstrate the inhibitory activities of P5 and DcP5 on the colony formation of DU145 cells. B, Quantification of the inhibitory activities of P5 and DcP5 on the colony formation of DU145 cells (*P < .05, **P < .01, ***P < .001). C, The growth‐inhibition activities of DcP5 in wild type and FGFR2‐knockdown DU145 cells (*P < .05, **P < .01, ***P < .001, ****P < .0001). D, Typical images of DU145‐based xenograft in null mice with different doses of DcP5 treatment. E, Typical images of DU145‐based tumors with different doses of DcP5 treatment. F, Quantification of tumor volume in DU145‐based xenograft exposed to different doses of DcP5. G, Quantification of tumor weight in DU145‐based xenograft exposed to different doses of DcP5 (columns with the same letter refer to *P *> .05, while columns with different letters refer to *P *< .05)

Considering DcP5 demonstrated stronger stability and binding affinity to FGFR2, we hypothesized that DcP5 would show better anticancer activity than P5. To test this, the growth‐inhibition rate was measured in DU145 cells, and the result demonstrated that DcP5 exhibited stronger inhibition than P5 at concentrations of 6.25 and 12.5 μM (Figure 4D). The inhibition of colony formation of DcP5 was performed at a concentration of 12.5 μM by using the same concentration of P5 peptide as control. The typical images of the cell colonies and clone numbers exposed to DcP5 and P5 groups are demonstrated in Figures 5A and 5B, respectively. According to the statistical analysis, cells exposed to DcP5 showed 20% less colonies than cells exposed to P5 (*P *< .05), although they were both significantly less than the control group (*P *< .01 and *P *< .001). Our results indicated that cyclization significantly improved the anticancer activity of P5 peptide in vitro.

To investigate the importance of FGFR2 in the inhibitory activity of DcP5 on the growth of DU145 cells, FGFR2 was knocked down by employing shRNA. The results demonstrated that the growth inhibition induced by DcP5 significantly decreased in the FGFR2‐knockdown DU145 cells compared to the wild type cells (Figure 5C).

To test the anticancer activity of DcP5 in vivo, xenograft model was employed by subcutaneously planting DU145 cells. Mice with 0.4‐0.5 cm^2^ subcutaneous tumor were randomly selected and assigned to each experimental group. Orthotopic injection was performed daily for a week with different doses of DcP5 and P5. On Day 14, tumors as well as normal organs were sampled for further analysis. As shown in Figure 5D‐G, DcP5 significantly decreased the tumor size in dose‐dependent manner. This finding indicated DcP5 as an excellent candidate for cancer therapy in vivo.

To determine the toxicity of DcP5, the organ tissues were collected and subjected to H&E staining. Figure S3B demonstrated that the morphology of heart, liver, spleen, lung, and kidney remained normal after treatment with DcP5. In addition, we examined changes in body weights of the mice in various dosing regimens and found that there was little difference in body weight between the individuals in the P5, DcP5, AZD4547, and blank groups (data not shown). These results indicated that DcP5 had no obvious acute toxic damage to mice.

### 2.6 P5 and DcP5 peptides inhibit tumor growth with the involvement of RTK‐signaling inactivation

**Figure 6:**
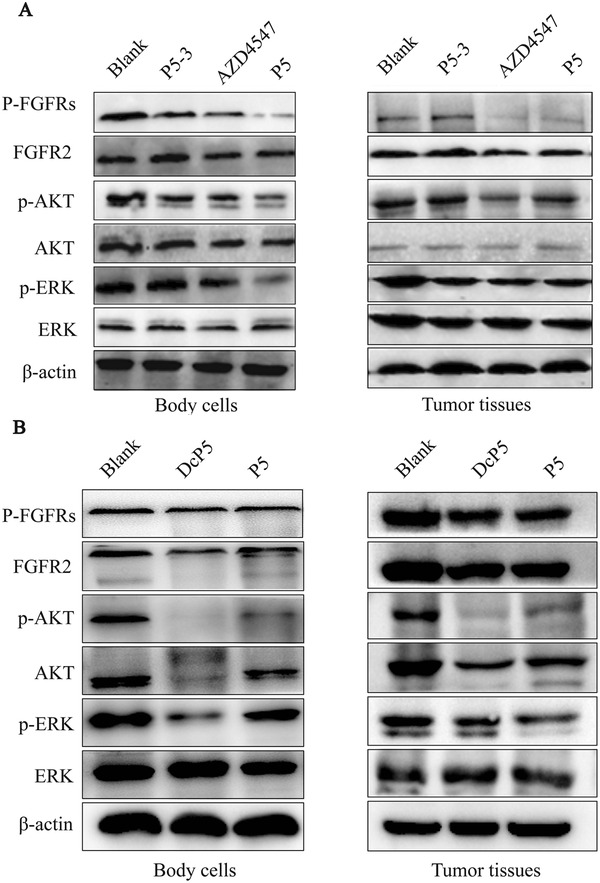
Potential mechanisms of the anticancer activities of P5 and DcP5. Western blotting of the phosphorylation levels of FGFR2, AKT, and ERK1/2 proteins in tumors and normal tissue of null mice exposed to P5 (A) and DcP5 (B)

To investigate the activities of DcP5 and P5 on the RTK‐signaling pathways, which are the downstream pathways of FGFR2, we performed Western blot analysis on tumor cells and normal cells surrounding the tumor to evaluate the phosphorylated FGFR2, AKT, and ERK1/2. Figure 6 shows that the phosphorylation of FGFR2, AKT, and ERK1/2 in the DcP5, P5, and AZD4547 groups was reduced. Moreover, there was no significant change in the levels of phosphorylation of these proteins in the P5‐3 and blank groups, thus demonstrating that the phosphorylation downregulation effect is epitope‐dependent.

## 3 DISCUSSION

FGFR2 has also been proposed as a potential therapeutic target in various malignancies because its expression levels were closely associated with the grade of malignancy and prognosis.^18^, ^19^ Some molecules have been designed to inhibit the activation of FGFR2, however, their performances are not satisfactory enough in clinical and preclinical studies.^20^ In our current study, we developed FGFR2‐targeted inhibitory peptide P5, which has been at the same time upgraded to a more stable and effective form (DcP5) through cyclization. Our results highlight that both DcP5 and P5 are potential anticancer agents and cancer‐targeted peptides for FGFR2‐overexpressed malignancies.

The peptide‐based drugs are receiving increasing attentions for their therapeutic potentials.^21^ Over 60 peptide‐based drugs have been approved by FDA for therapeutic use for various diseases and are approaching clinical application at a steady pace.^22^ One of the major diseases that benefits from peptide‐based drugs is metabolic disorder.^23^ For instance, one of the best‐known peptide drugs, insulin was first discovered in the 1920s and is still being widely used in type I diabetic patients. Although many chemical molecules have been discovered for controlling glucose in patients’ blood that are simpler to use, such as oral administration, insulin is still the best agent that improves the life qualities of these type I diabetic patients, demonstrating the powerful therapeutic potential of peptide‐based drugs.^24^, ^25^ In this study, we developed a potential therapeutic peptide that targets FGFR2 and examined its biological activities in vitro and in vivo, as well as toxicity. Although currently some molecules have been proved to inhibit the activation of FGFR2 effectively, the metabolism and clearance of peptides are naturally safer than these synthetic chemicals. Therefore, our discoveries provide two FGFR2‐silencing peptides, which are potential candidates for diseases caused by FGFR2 overexpression.

According to statistical studies, another major application area of peptide‐based drugs is oncology therapy.^26^ Currently, the majority of anticancer peptides belong to cytotoxicity‐based agents. These kind of peptides were usually first discovered as antimicrobial peptides, which later on were proved to demonstrate anticancer activities through targeting the cell membrane, however, they do not damage the cancer cells only.^27^ For instance, SVS‐1 is an 18‐residue peptide that kills cancer cells through induction of pore formation on the cells.^28^ d‐K_6_L_9_ peptide induces necrosis in prostate cancer cells via depolarizing their cell membrane.^29^ Recently, researchers have found another kind of anticancer peptides that target cancer through recognition of receptors specifically expressed on cancer cells or are crucial for survival of cancer cells, but are not on normal cells.^30^ For instance, buserelin is one of the peptide‐based LHRH agonist for prostate cancer therapy by blocking the signaling induced by LHRH, which is critical for prostate cancer, but not normal tissues. Thus, it causes minimal toxicity toward normal cells.^31^ FGFR2 is overexpressed on many different kinds of cancer cells and is crucial for their proliferation.^32^, ^33^ Inhibition of FGFR2 signaling is a potential strategy for cancer therapy.^34^, ^35^ In our current study, we developed P5 and DcP5 peptides as competitors for FGF2, and evaluated its anticancer activities in vitro and in vivo. In this manner, P5 and DcP5 neutralize FGF2/FGFR2 signaling, resulting in the inactivation of the downstream signaling, such as AKT and ERK phosphorylation, which are well known to be oncogenic signaling. Our study contributed two potential anticancer peptides for malignancy therapy.

Interestingly, the sequence of P5 peptide is exactly the same as one of the bFGF‐degraded debris that were identified in vivo in physiological conditions.^36^ This might be one of the negative feedback regulatory mechanisms of bFGF signaling. Once the concentration of bFGF is overloads, proteinases could be activated to control the overactivation of FGFR2 by degrading bFGF. In this manner, the amount of bFGF protein decreases, and some of the debris, such as the one with the same sequence of P5 peptide, even compete with the full‐length bFGF, resulting in the decrease of FGFR2 signaling quickly. This might be one of the in vivo cooling down mechanisms of bFGF/FGFR2 signaling. Considering that our P5 peptide shares the same sequence with the debris of bFGF, we hypothesized that P5 peptide would show minimal toxicities toward normal tissues, and our results successfully supported this hypothesis. These findings prove the selectivity of P5 peptide for cancer therapy. Moreover, we also put forward a potential negative feedback regulation of bFGF/FGFR2 signaling, which is also a potential reason for the low toxicity of P5 peptide toward normal tissues.

Generally, peptide drugs exhibit excellent activity, safety, and biocompatibility; however, the poor stability is a drawback. Investigators have put forward several strategies to improve the stabilities of peptide drugs, such as the application of unnatural amino acids, modification at the N‐terminus or C‐terminus of the peptide, and cyclization of the peptide.^37^ Among these, cyclization is one of the most powerful and simplest approaches. Through cyclization, peptides are constricted into a more stable and defined conformational form. One of the best‐known examples is the RGD peptide. The cyclized RGD demonstrated enhanced stability and integrin‐binding affinity compared to the linearized RGD.^38^, ^39^ In our current study, we cyclized P5 peptide and developed a DcP5 with improved stability and anticancer activities in vitro and in vivo. Our findings highlight an improved FGFR2‐targeting cyclic peptide for cancer therapy.

In summary, we developed a peptide (P5) that has the same primary structure with a naturally degraded debris of bFGF, to limit the activity of FGFR2 through competitive binding with bFGF. The binding between P5 and FGFR2 is dependent on the Gln4 and Glu6 of P5. Through the specific inhibition of FGFR2 and its downstream signaling, P5 demonstrated excellent anticancer activities and negligible toxicity in vitro and in vivo. The stability and binding affinity of P5 was further improved through cyclization. The cyclized P5 (DcP5) demonstrated even stronger anticancer activities in vivo. Our results suggest P5 and DcP5 as potential candidates for future cancer therapeutics.

## 4 MATERIALS AND METHODS

### 4.1 Peptide design and synthesis

The peptide binding to FGFR2 was analyzed based on the reported crystal structure of bFGF (PDB ID: 4OEE),^40^ and peptide‐binding simulation method was employed to analyze the potential interaction site. The potential binding between the designed peptides and FGFR2 was evaluated through the ZDOCK system (Discovery Studio 4.1, Accerlys Ltd., San Diego, CA). The clustering poses were predicted according to ligand positions. The peptide‐binding pose that occupied the predicted binding cavity in the extracellular domain of FGFR2 was chosen for further analysis. After obtaining the FGFR2‐peptide binding model, optimization and restrained minimization were performed for further analysis to identify critical interactions. Based on the information of nonbonding interactions, electrostatic interactions, and hydrogen bond networks between FGFR2 and peptides, the model was further manually viewed.

A set of peptide candidates was designed with manual inspection, synthesized with a chemical dehydration reaction, and verified with MS. To screen the candidates, an in vitro binding assay using isothermal titration calorimetry (ITC) was performed against the FGFR2 protein using AutoiTC200 (MicroCal, MA). After the positive candidate was confirmed by ITC, three analog peptides with different mutant amino acids were designed to verify the active site previously determined. The binding affinity of each mutant analog to FGFR2 was measured using ITC and compared with the positive candidate.

### 4.2 Cell culture and proliferation

The cell lines, including DU145 (human prostate cancer cells, American Type Culture Collection/ATCC, Manassas, VA), LNCaP (human prostate cancer cells, ATCC, Manassas, VA), KYSE30 (human esophageal squamous cell carcinoma cells, ATCC, Manassas, VA), KYSE510 (human esophageal squamous cell carcinoma cells, ATCC, US), L02 (human normal liver cells, ATCC, Manassas, VA), and MCF‐7 (human breast cancer cells, ATCC, Manassas, VA) were cultured in DMEM medium (Thermo Gibco, Waltham, MA) with 10% FBS (Biological Industries, Israel). The SGC7901 (human gastric cancer cells, ATCC, Manassas, VA) cells were maintained in RPMI‐1640 medium (Thermo Gibco, Waltham, MA) with 10% FBS. All these cell lines were cultured at 37°C in a 5% CO_2_ cell incubator (Bio‐Rad, Hercules, CA). Subculture of these cell lines was employed with 0.25% trypsin (Thermo Gibco, Waltham, MA).

The cells were seeded at a density of 30 000 cells/mL based on cell counting performed with a cell counting plate. Different concentrations of the P5 peptide were added to the cultural medium, followed by a 24‐hour incubation at 37°C in a 5% CO_2_ cell incubator (Bio‐Rad, Hercules, CA). Five replicates were set up for each experimental group.

Cell proliferation was evaluated using the Enhanced CCK‐8 cell proliferation and toxicity test kit (Meilunbio, China) according to the product specifications. Finally, the OD450 nm and OD630 nm were measured with an iMark dual wavelength microplate reader (Bio‐Rad, Hercules, CA), and the ratio of OD450 nm/OD630 nm was calculated.

Cell colony formation was also employed to evaluate the effect of P5 and DcP5. The DU145 cells were seeded in six‐well plates (500 cells/well). The cultural supernatant was removed at 48 hours posttreatment, followed by washing with PBS three times. The cells were then fixed with 4% paraformaldehyde and air dried. Crystal violet staining was performed using solution (Sangon, China) as per the instructions. Then, photographs were taken, and spot amounts were calculated with an Invitrogen Countess II FL (Thermo, Waltham, MA) cell counter.

### 4.3 Binding‐affinity analysis

The binding affinity was analyzed under fluorescent microscope. GFP‐labeled DU145 cells were cultured to a density of 50%, followed by 4% paraformaldehyde fixation. After treated with 0.2% Triton X‐100, the cells were then blocked with 5% BSA. The incubation was performed by different treatments and SA‐PE‐TRITC was used as a fluorescent indicator; briefly, the FGFR2 mAb group (Abcam [Cambridge, MA] followed by PE‐TRITC‐labeled goat antimouse IgG for color development), and the blank group (equal amounts of PBS followed by SA‐PE‐TRITC as a fluorescent indicator were added). After adding the biotin‐labeled P5 peptide, biotin‐labeled P5‐3 peptide or anti‐FGFR2 mAb, the cells were incubated at 4°C overnight. After washing five times with PBST, the TRITC‐labeled streptavidin regent or TRITC‐labeled secondary antibody was introduced for another incubation at 25°C for 30 minutes in dark. Finally, the DAPI staining reagent was employed to stain the nucleus for 10 minutes in dark, followed by washing five times with PBS. After the addition of ProLong Antifade mounting medium (Thermo, Waltham, MA), a confocal microscope (OLS4100, Japan) was used for observation.

### 4.4 Cyclization optimization

In our study, peptide cyclization was achieved by adding two cysteine residues to each end of the peptides. Cysteine is a chiral molecule. The natural cysteine is l‐cysteine, and a report has shown that introduction of unnatural d‐cysteine can theoretically improve the stability of the cyclic peptide in vivo.^41^, ^42^, ^43^ Therefore, we designed and synthesized two cyclic peptides, DcP5 and LcP5, with two l‐cysteines or d‐cysteines at each end, respectively. The amino acid polymers of DcP5 and LcP5 were synthesized via the same organic chemistry as the P5 peptide. The cysteine‐ending peptides were spontaneously cyclized at 37°C in PBS (pH = 9.0) for 12 hours. The molecular weight and purity of the two polypeptides were then identified by MS as described in Section 2.1. Then, the binding affinity of these two cyclic peptides to FGFR2 was measured by ITC as described in Section 2.1. The cyclic peptide with the stronger signal was selected to undergo intracellular interaction as determined by the IFA method described in Section 2.3, and this result was compared with the result from the liner P5 peptide. The effect on cell proliferation inhibition of both the cyclic peptide and the liner peptide was determined with the cell colony formation assay described in Section 2.2 with final concentration of each peptide of 12.5 μM. The results of the cyclic peptide and liner peptide were aggregated together and further contrasted with each other to determine effect of the change after the cyclization optimization.

### 4.5 Xenograft

The specific pathogen‐free (SPF) level experimental male nude mice were purchased from the Animal Center of Guangdong Province (China), and the strain was BALB/C‐nu/nu. The animal experiments were conducted by the Animal Experimental Center of Jinan University (China) according to the National Institutes of Health guide for the care and use of Laboratory animals. The treatment and experimental procedure met the requirements of animal ethics.

DU145 cells with a density above 90% were harvested in to a microtube. The cell concentration was adjusted to 1 × 10^7^ cells/mL, and two million cells were injected under the right arm of each individual. After injection, the tumors grew to a size of approximately 200 mm^3^ in the following days. In terms of experimental grouping, there were five experimental groups; namely, the blank control group (PBS), the P5‐3 group (50 mg/kg dose of biotin‐P5‐3), the P5 group (concentration gradient of 10‐25 mg/kg), the DcP5 group (concentrations of 10, 25, and 50 mg/kg dose of biotin‐DcP5 peptide), and the positive control group (FGFR2‐targeted antineoplastic drug AZD4547 with a dose of 6.25 mg/kg). Animal models were randomly selected and assigned to each experimental group, and compounds were administered by intratumoral injections. The dosing regimen was one injection a day for 7 days. The length and width of the tumor were measured using a Vernier caliper once a day, and the animal's weight was recorded once a day from the beginning of the experiment. On Day 14, blood samples of each animal were collected, the tumor tissue was separated and weighed, and internal organs were collected. The tumor volume was calculated according to the formula *V* = *ab* ^2^/2 (*a* refers to the length of the tumor, and *b* refers to the width).

### 4.6 Hematoxylin and eosin (H&E) staining

The cell morphology was demonstrated in the tissue sections with H&E staining. Briefly, the connective tissue around the parenchyma organ was removed and washed three times in PBS, and the tissues were fixed with 10% formalin solution for 24 hours, followed by gradient ethanol dehydration. The tissues were then subjected to paraffin embedding. Sections of 4 μm thickness were prepared for H&E staining. H&E staining was performed according to the standard protocol of the H&E staining kit (Solarbio, China).

### 4.7 Concentration of P5 and DcP5 in peripheral blood

Serum in the blood was separated and used to perform biotin quantitation with a Biotin Quantitation assay kit (MyBioSource, San Diego, CA) to calculate the concentration of the biotin‐peptide complex in the blood sample. Serum samples from the blank group were used as background controls, and serum of the 25 mg/kg‐dose P5 group and 25 mg/kg‐dose DcP5 group was examined in this step.

### 4.8 Real‐time qPCR

The mRNA level of FGFR2 in different cell lines was measured by real‐time quantitative polymerase chain reaction (RT‐qPCR). Briefly, mRNA of cells was extracted by using TRIzol‐based method. Reversed transcription was performed by the SuperScript Reverse Transcriptases kit (Invitrogen) to obtain cDNA. The expression of FGFR2 was presented as the relative expression level between different cell lines by using the house‐keeping gene β‐actin as control.

### 4.9 Western blot assay

Cell lysates were subjected to SDS‐PAGE for Western blotting. Briefly, cells were lysed with RIPA buffer (Thermo, Waltham, MA), followed by heat denaturation in 1X Laemmli SDS buffer (Sigma, St Louis, MO). For SDS‐PAGE 100 μg proteins were loaded to each lane. After electrophoresis, the proteins were transferred onto PVDF membranes, followed by blocking in 5% nonfat milk. The blocked membranes were then overnight probed with primary antibodies (1:5000 in primary antibody dilution buffer), including antiphosphorylated FGFRs (Abcam, Cambridge, MA), anti‐FGFR2 (Abcam, Cambridge, MA), antiphosphorylated AKT (Abcam, Cambridge, MA), anti‐AKT (Abcam, Cambridge, MA), antiphosphorylated ERKs (Abcam, Cambridge, MA), anti‐ERK1/2 (Abcam, Cambridge, MA), and anti‐beta‐actin (Abcam, Cambridge, MA). The membranes incubated with primary antibodies were then washed with TBST before being incubated with peroxidase‐conjugated secondary antibody (1:20 000 in secondary antibody dilution buffer). After another round of TBST washing, the PVDF membranes were developed with ECL reagent (Millipore, MA) in the Gel‐Doc imaging system (Bio‐Rad, Hercules, CA). The bands were quantified using Image Lab Software 6.0 (Bio‐Rad, Hercules, CA).

### 4.10 FGFR2 knockdown

The shFGFR2 plasmid was purchased from Sigma (St Louis, MO) (TRCN0000218493). One microliter Lip3000 (Lipofectamine 3000, Invitrogen, Carlsbad, CA) was mixed with 100 ng plasmid in nonserum DMEM medium before being added into the cultural DU145 cells. The cells were incubated with the Lip3000/plasmid solution for 6 hours, followed by DMEM with 10% FBS incubation. The transfected cells were then ready for growth‐inhibition experiment and RNA extraction for qPCR.

### 4.11 Statistical analysis

One‐way ANOVA and two‐way ANOVA were used for multiple comparisons, while two‐tailed Student's *t*‐test was employed for single comparison (*P *< .05). Analyses were performed using GraphPad Prism 7. The results are presented as the mean ± SD.

## CONFLICT OF INTEREST

The authors declare that there is no conflict of interest.

## ETHICS STATEMENT

This study was approved by the research ethics committee of Jinan University

## AUTHOR CONTRIBUTIONS

Conception and design, data analysis and interpretation, drafting the article, revising the article, and final approval of the version to be published: Yibo Zhang. Design, experimentation, data acquisition, and analysis: Man Ouyang, Hailong Wang, and Wenhua Guang. Experimentation and data acquisition: Bihui Zhang, Zhixin Li, and Zijian Su. Technical support: Ruiwu Liu and Xiaocen Li. Conception and design: Tsung‐Chieh Shih. Data analysis and drafting the article for intellectual content: Jieqiong Cao and Qiling Meng. Conception and design: Jinshao Ye and Feng Liu. Project supervision, conception and design, data analysis and interpretation, and final approval of the version to be published: An Hong and Xiaojia Chen.

## Supporting information

Supporting Information

## Data Availability

The data that support the findings of this study are available from the corresponding author upon reasonable request.
