# Supplementary material for: A cyclic peptide retards the proliferation of DU145 prostate cancer cells in vitro and in vivo through inhibition of FGFR2
Source: MedComm (2020). 2020 Dec 14;1(3):362–75. doi: 10.1002/mco2.48 (PMC8491194; doi:10.1002/mco2.48)
Supplement: Supplementary file 1 — Supporting Information [file MCO2-1-362-s001.pdf]

Supplemental Figures:

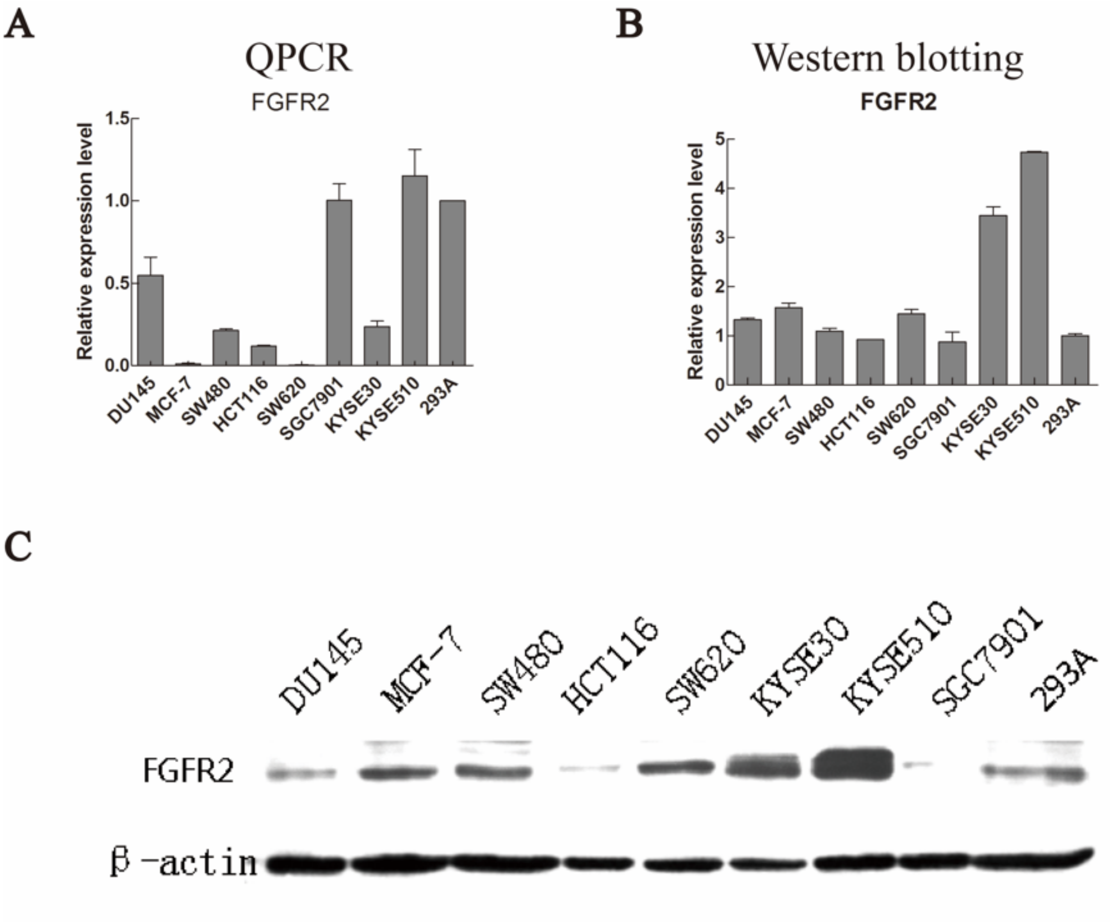

Figure S1 Expression levels of FGFR2 in different cell lines. The mRNA level (A) and protein level (B-C) of FGFR2 in different cell lines.

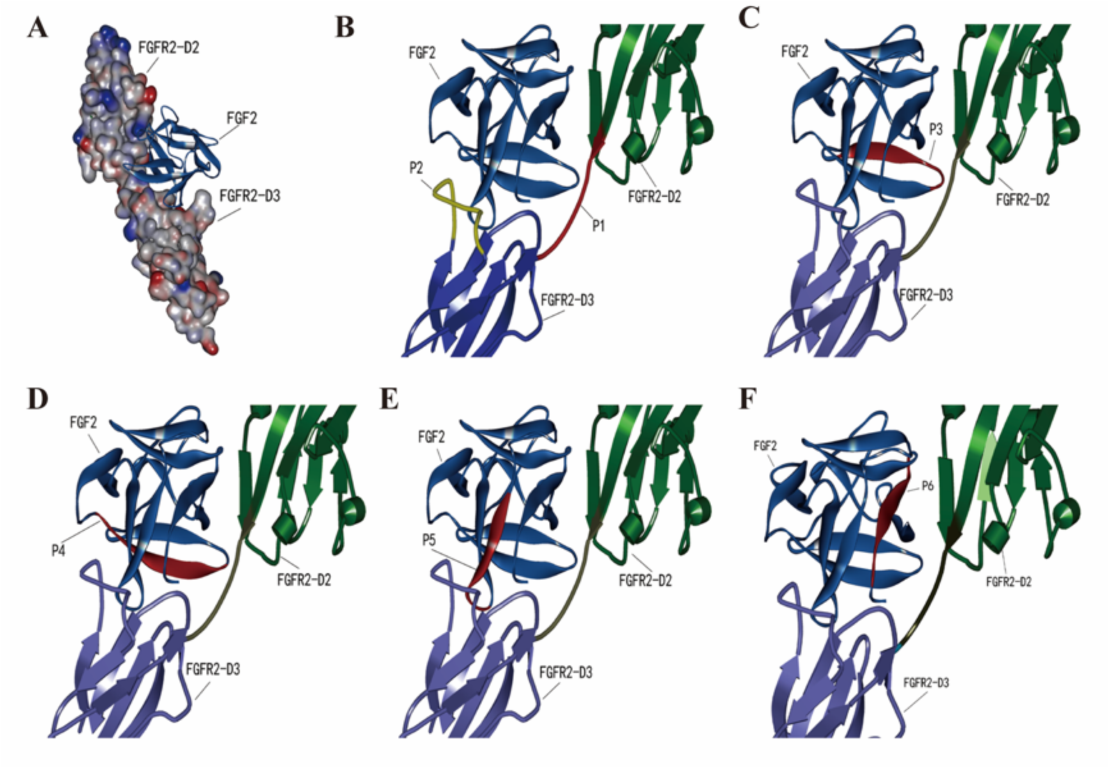

Figure S2 Binding sites of FGF2 and the designed epitope peptides with FGFR2. (A) Binding site of FGF2 with FGFR2; (B) Binding site of P1 with FGFR2; (C) Binding site of P3 with FGFR2; (D) Binding site of P4 with FGFR2; (E) Binding site of P5 with FGFR2; (F) Binding site of P6 with FGFR2.

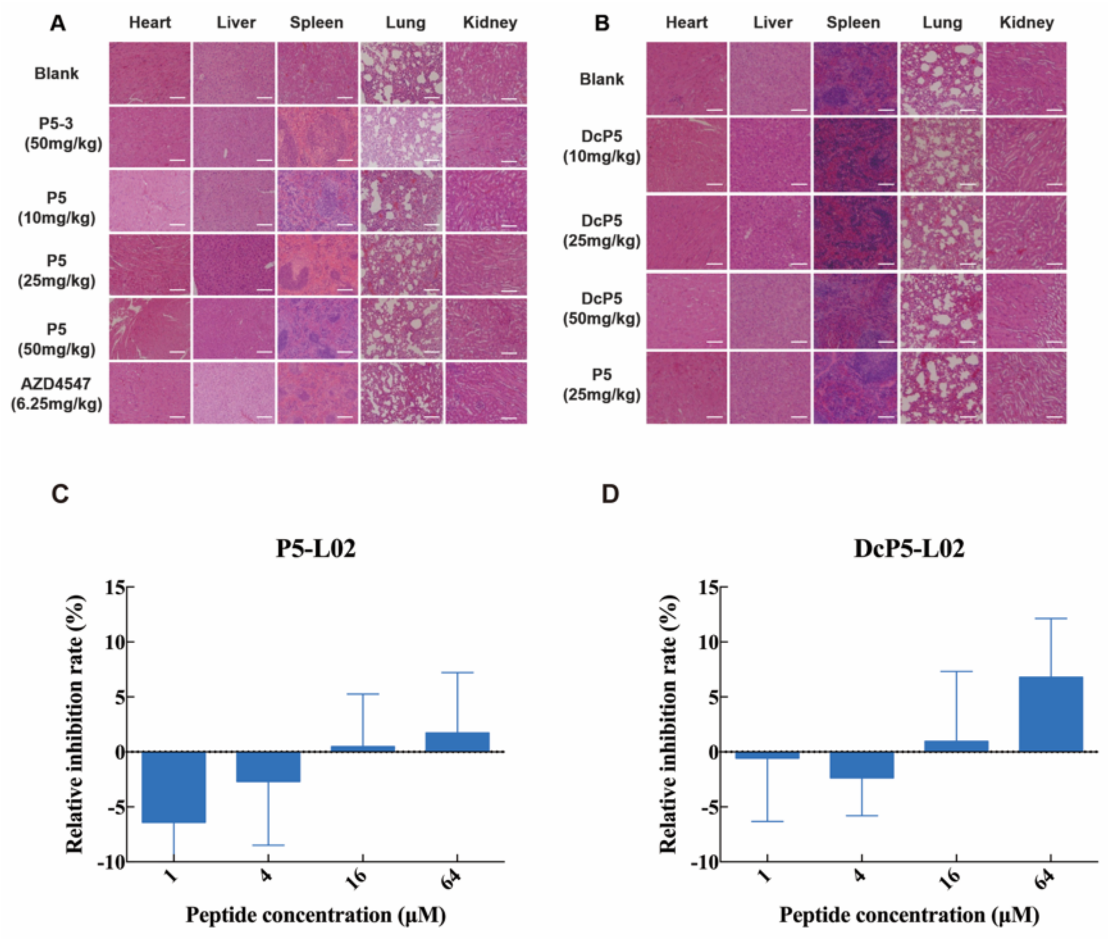

Figure S3 HE staining of the major organ tissues of null mice and normal cell line exposed to P5(A) and DcP5 (B) and toxicity of P5 and DcP5 towards L02 cells (C).

A

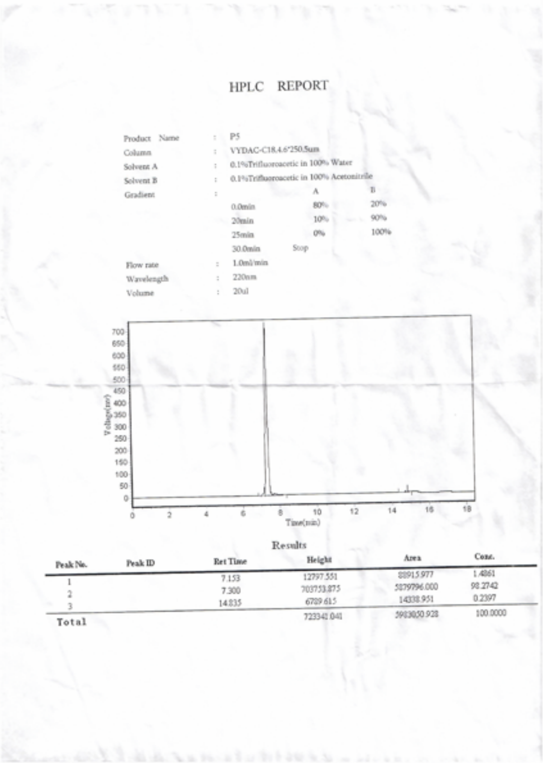

HPLC REPORT

Product Name : P5
Column : VYDAC-C18.4.6*250.5um
Solvent A : 0.1%Trifluoroacetic in 100% Water
Solvent B : 0.1%Trifluoroacetic in 100% Acetonitrile
Gradient :

	A	B
0.0min	80%	20%
20min	10%	90%
25min	0%	100%
30.0min	Stop	

Flow rate : 1.0ml/min
Wavelength : 220nm
Volume : 20ul

Results

Peak No.	Peak ID	Ret Time	Height	Area	Conc.
1		7.153	12797.551	88915.977	1.4861
2		7.300	703753.875	5879796.000	98.2742
3		14.835	6789.615	14338.951	0.2397
Total			723341.041	5983050.928	100.0000

B

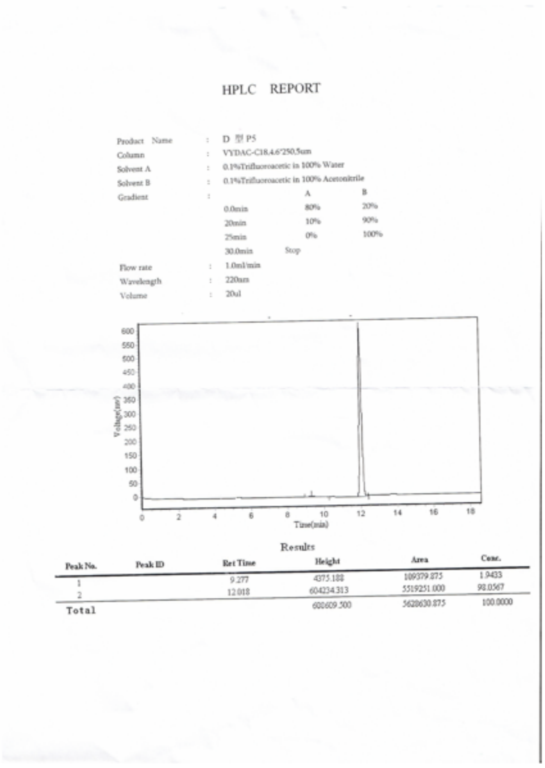

HPLC REPORT

Product Name : D 型 P5
Column : VYDAC-C18.4.6*250.5um
Solvent A : 0.1%Trifluoroacetic in 100% Water
Solvent B : 0.1%Trifluoroacetic in 100% Acetonitrile
Gradient :

	A	B
0.0min	80%	20%
20min	10%	90%
25min	0%	100%
30.0min	Stop	

Flow rate : 1.0ml/min
Wavelength : 220nm
Volume : 20ul

Results

Peak No.	Peak ID	Ret Time	Height	Area	Conc.
1		9.277	4375.188	109379.875	1.9433
2		12.018	604234.313	5519251.000	98.0567
Total			608609.500	5628630.875	100.0000

Figure S4 Purity of P5 (A) and DcP5 (B) measured by HPLC.

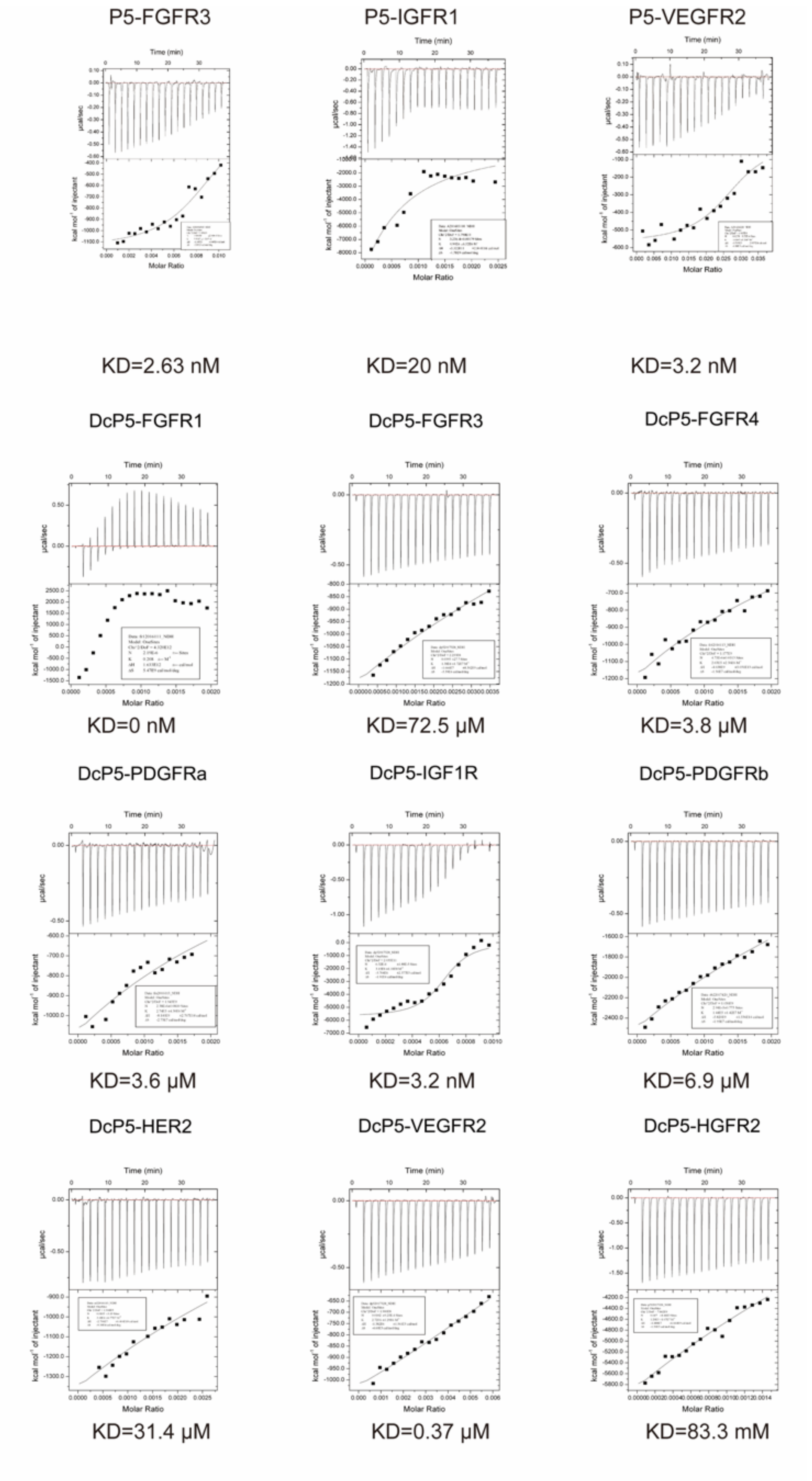

Figure S5 Binding affinity of P5 and DcP5 to other RTK members.
